# Unsupervised Hyperspectral Band Selection Using Spectral–Spatial Iterative Greedy Algorithm

**DOI:** 10.3390/s25185638

**Published:** 2025-09-10

**Authors:** Xin Yang, Wenhong Wang

**Affiliations:** College of Computer Science, Liaocheng University, Liaocheng 252000, China

**Keywords:** band selection, hyperspectral remote sensing images, iterative greedy algorithm, spectral–spatial information, local search

## Abstract

Hyperspectral band selection (BS) is an important technique to reduce data dimensionality for the classification applications of hyperspectral remote sensing images (HSIs). Recently, searching-based BS methods have received increasing attention for their ability to select the best subset of bands while preserving the essential information of the original data. However, existing searching-based BS methods neglect effective exploitation of the spatial and spectral prior information inherent in the data, thus limiting their performance. To address this problem, in this study, a novel unsupervised BS method called Spectral–Spatial Iterative Greedy Algorithm (SSIGA) is proposed. Specifically, to facilitate efficient local search using spectral information, SSIGA conducts clustering on all the bands by employing a *K*-means clustering method with balanced cluster size constraints and constructs a *K*-nearest neighbor graph for each cluster. Based on the nearest neighbor graphs, SSIGA can effectively explore the neighborhood solutions in local search. In addition, to efficiently evaluate the discriminability and information redundancy of the solution given by SSIGA using the spatial and spectral information of HSIs, we designed an effective objective function for SSIGA. The value of the objective function is derived by calculating the Fisher score for each band in the solution based on the results of the superpixel segmentation performed on the target HSI, as well as by computing the average information entropy and mutual information of the bands in the solution. Experimental results on three publicly available real HSI datasets demonstrate that the SSIG algorithm achieves superior performance compared to several state-of-the-art methods.

## 1. Introduction

Hyperspectral remote sensing is an advanced Earth observation technology. It uses a hyperspectral imaging spectrometer to simultaneously capture images of ground objects across dozens or even hundreds of spectral bands, thereby generating hyperspectral remote sensing images (HSIs) that contain rich spatial and spectral information about land cover. Recently, HSIs have been widely used in various fields such as target detection [1], land cover classification [2,3], environmental monitoring [4,5], and precision agriculture [6,7]. However, the large number of bands included in the HSI results in high data dimensionality. On the other hand, the high correlation between bands leads to a large amount of information redundancy [8,9,10]. This not only causes a computational burden [11,12] but also leads to the Hughes phenomenon in hyperspectral classification tasks [13,14]. An effective way to address this problem is to reduce the dimensionality of HSIs.

Hyperspectral dimensionality reduction primarily falls into two categories: feature extraction and band selection. In recent years, deep learning-based feature extraction has become a prominent approach. For instance, various convolutional neural network (CNN)-based and transformer-based networks have demonstrated powerful capabilities in learning discriminative spectral–spatial representations. Notable examples include the multiscale neighborhood attention transformer (MSNAT) [15], the 3D spectral–spatial Mamba (3DSS-Mamba) [16], and the Mamba-in-Mamba (MiM) framework [17]. These models leverage hierarchical convolutions, attention mechanisms, or state-space modeling to capture local and global dependencies, thereby achieving superior classification accuracy compared to traditional machine learning methods. The advantage of such feature extraction methods lies in their ability to automatically learn high-level semantic features from original data, reducing the reliance on manual feature engineering. However, they typically convert the original spectral bands into a latent feature space, which weakens the physical interpretability of the results. In addition, they usually require a large amount of labeled data and computational resources [17].

By contrast, band selection (BS) directly identifies a subset of informative spectral bands from the original HSI cube without altering their physical meanings [18,19,20]. This not only reduces data redundancy and computational cost, but also preserves intrinsic spectral characteristics, making the results more interpretable and physically meaningful for remote sensing applications [21]. Based on the availability of labeled samples during the training phase, band selection methods are generally categorized as supervised [22], semi-supervised [23], and unsupervised methods [24,25]. Given the complexities involved in accurately labeling HSIs, unsupervised methods are often preferred in practical applications. Unsupervised band selection methods can be broadly divided into ranking-based methods [26,27,28], clustering-based methods [29,30], and searching-based approaches [31,32,33].

Ranking-based methods assign ranks to spectral bands based on predefined criteria and subsequently select the top-ranked bands to constitute a band subset. Although this method is effective in identifying promising subsets, it tends to ignore correlations between bands, which may lead to redundancy between selected bands [34]. Clustering-based methods treat each spectral band as an individual data point and group spectral bands with similar characteristics into a cluster. Then, from each group, a representative spectral band is selected according to specific evaluation criteria to form the final subset of spectral bands [35,36]. This strategy effectively decreases the correlation between bands, thus reducing the redundancy of the selected bands. However, since the selected bands come from different clusters, clustering-based methods can overemphasize the individual importance of the bands while neglecting the overall performance of the selected band subset [37].

Searching-based methods commonly regard band selection as a discrete combinatorial optimization problem, given their effectiveness in exploring large solution spaces [38,39,40,41]. Recently, various meta-heuristic optimization algorithms, including the gray wolf optimization algorithm [42], artificial bee colony algorithm [43], and heterogeneous cuckoo search algorithm [44], have been applied to BS tasks. In [42], the authors proposed a band selection method based on the gray wolf optimization algorithm, utilizing the hunting behavior of wolves as a search mechanism to explore the optimal band subset. In addition, category separability was incorporated into the population initialization process, effectively mitigating the risk of converging to local optima. In [43], the band selection problem is transformed into a multi-task optimization framework where clustering techniques are combined with an artificial bee colony algorithm to identify multiple optimal band subsets of varying sizes. In [44], the researchers proposed an unsupervised BS method via the enhanced heterogeneous cuckoo search algorithm integrated with a matched filter. This method uses a mapping method based on neighborhood band grouping to reduce the similarity between selected bands. Although existing searching-based BS methods have demonstrated better performance, these methods ignore the spatial–spectral properties in real HSIs, and thus their performance is limited. Therefore, it remains a challenge to use the spatial–spectral prior information in real HSIs to design effective algorithmic search strategies and objective functions that can guide searching-based BS methods to find band combinations with high discriminability and low information redundancy.

To address these problems, a novel unsupervised hyperspectral band selection method called Spectral–Spatial Iterative Greedy Algorithm (SSIGA) is proposed in this paper. SSIGA aims to improve the efficiency of band selection by incorporating clustering and superpixel segmentation techniques into IGA, thereby exploiting the spatial and spectral information of the data. Specifically, to facilitate efficient local search using spectral information, SSIGA employs a *K*-means clustering method with balanced cluster size constraints to conduct clustering on all the bands. Then, based on a group of *K*-nearest neighbor graphs (*K*-NNGs) constructed for each cluster, SSIGA can exploit efficient neighborhood solutions in local search. To efficiently evaluate the discriminability and redundancy of the information of the band subset given by the solution, an effective objective function for SSIGA is designed. To evaluate the value of the objective function, we not only calculate the Fisher score for each band in the solution based on the result of performing superpixel segmentation on the HSI data, but also compute the average information entropy and mutual information of the bands given by the solution. Experimental results on three publicly available real HSI datasets demonstrate that SSIGA achieves superior performance compared to several state-of-the-art methods. The contributions of this article include the following.

(1) A novel unsupervised Spectral–Spatial Iterative Greedy Algorithm-based band selection method is proposed. To the best of our knowledge, this is the first time that the IG algorithm is used to solve the band selection problem.
(2) By conducting clustering on all bands and constructing a *K*-NNG for each cluster, our proposed SSIGA enables efficient neighborhood solution construction, which helps to facilitate efficient local search using spectral information.
(3) We designed an effective objective function that can evaluate the quality of the solution by calculating the average information entropy of all the bands in the solution and the average mutual information between the bands, as well as the Fisher score of each band. Experimental results show that SSIGA outperforms several state-of-the-art methods.

The rest of this paper is structured as follows: Section 2 details the proposed SSIGA method. Then, we present the experimental results and related analyses in Section 3. Finally, Section 4 concludes this study.

## 2. Method

### 2.1. Iterative Greedy Algorithm

IGA was first proposed for solving large ensemble coverage problems in 1995 [45]. As a simple and effective heuristic algorithm, IGA solves an optimization problem based on a single solution [46]. Essentially, IGA can be regarded as a special kind of local search method [47]. It solves the problem by continuously exploring better solutions in the neighborhood of the current solution and adopts some perturbation strategies to avoid local optimal solutions. Unlike traditional local search methods, IGA has two unique perturbation phases, namely the destruction and construction phases.

Specifically, through the destruction strategy candidate solutions are broken and then new solutions are reconstructed via construction heuristics. Through the perturbation stage, IGA improves the quality of the solution by searching the neighborhood of the current solution to identify the local optimum. Finally, a specific acceptance criterion is employed to determine how the algorithm selects a new solution at each iteration. Due to its simplicity and minimal parameter requirements, IGA is both efficient and easy to implement. Currently, IGA is a widely used heuristic approach for solving combinatorial optimization problems.

### 2.2. Iterative Greedy Algorithm Based on Simulated Annealing

The original IGA relies on greedy search, accepting only solutions better than the current one at each iteration. This makes it prone to falling into local optima. In contrast, the probabilistic acceptance mechanism of simulated annealing [48] allows for the acceptance of worse solutions with a certain probability, thereby increasing the possibility of escaping local optima. To improve its global optimization performance, IGA has recently been combined with simulated annealing  [49]. By introducing perturbations through simulated annealing after each local search stage, the search process of IGA is no longer confined to the current neighborhood, enabling IGA to explore a broader solution space and enhancing its global search capability.

Specifically, according to the simulated annealing algorithm, IGA accepts a new solution following specific rules  [50]. If a new solution given by the local search of IGA is superior to the current one, it is accepted unconditionally. Conversely, when the new solution is inferior, its acceptance is probabilistic, determined by a combination of factors. In other words, the acceptance probability decreases with the difference between the objective value of the new solution and that of the current solution, as well as with the current temperature. As the temperature decreases over iterations, IGA becomes less likely to accept inferior solutions. Specifically, the acceptance probability *P* of a new solution in IGA can be mathematically expressed as follows [51]:

(1) $$P=\exp\left(-\frac{\Delta F}{T}\right)$$

where ΔF denotes the difference between the new solution and the current solution in terms of the objective function value; *T* represents the current temperature in the simulated annealing process. As the algorithm progresses, the temperature *T* will gradually decrease, facilitating extensive exploration in the initial stages and fostering convergence towards optimal solutions in later stages. This approach significantly improves the algorithm’s capacity to avoid local optima, thereby enhancing the prospects of identifying superior solutions. The basic framework of IGA based on simulated annealing is outlined in Algorithm 1 [52,53].

**Figure 1:**
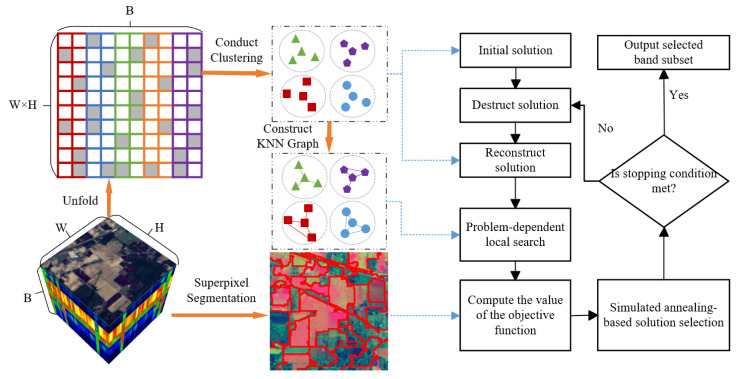
A schematic illustration of SSIGA.

In this section, we provide a detailed description of the proposed SSIGA method. Figure 1 gives a schematic illustration of SSIGA. As shown in Figure 1, SSIGA first performs superpixel segmentation on the original hyperspectral data, utilizing spatial consistency to aggregate pixels into local regions while preserving spatial structural information. In addition, the three-dimensional hyperspectral data is unfolded into a two-dimensional matrix, where clustering analysis is conducted based on spectral similarity, and a set of K-nearest neighbor graphs is constructed based on the results of clustering analysis according to the similarity between bands. During the optimization process of SSIGA, an initial band subset is first selected based on the clustering results. Then, a destruct–reconstruct strategy is applied, where a portion of the bands is randomly removed from the current solution and a new band subset is reconstructed. Next, a problem-dependent local search is performed on the current band subset to further optimize the band selection scheme. For each candidate solution, the objective function value is calculated based on spectral discriminability, information entropy, and mutual information to evaluate the classification performance, information content, and redundancy of the selected bands. Finally, to prevent the search from falling into local optima, SSIGA employs simulated annealing as the acceptance criterion, accepting or rejecting new solutions based on probability to achieve global optimization. The algorithm iteratively executes this optimization process and outputs the final optimal band subset once the stopping condition is met. The complete iterative optimization workflow is summarized in Algorithm 2.
**Algorithm 1** Framework of IGA.**Input:** 
The number of elements *q* that need to be removed from the current solution.**Output:** 
The best solution $\pi^{*}$.  1:$\pi_{0}\leftarrow$ Initialization;  2:π← Local Search($\pi_{0}$);  3:$\pi^{*}\leftarrow \pi$;  4:**while** termination criterion is not met **do**  5:    $\pi^{\prime }\leftarrow$ Destruction and construction(π,*q*);  6:    $\pi^{\prime }\leftarrow$ local search( $\pi^{\prime }$);  7:    **if** $F\left(\pi^{\prime }\right)<F\left(\pi \right)$ **then**  8:        $\pi \leftarrow \pi^{\prime }$;  9:        Update the optimal solution;10:    **else**11:        Randomly generate a number *r* from 0 to 1;12:        **if** $r<e^{-\frac{\Delta F}{T}}$ **then**13:            $\pi \leftarrow \pi^{\prime }$;14:        **end if**15:    **end if**16:    T←0.99×T;17:**end while**18:**return** $\pi^{*}$

**Algorithm 2** The algorithm of the SSIGA method.
**Input:** 
Hyperspectral images dataset *X*   Number of clusters *M*   Number of neighbors *K*   Number of superpixels *N*   Simulated annealing temperature *T*   Maximum number of iterations Iter**Output:** 
Selected band subset *Y*  1:Perform clustering and construct *K*-NNGs;  2:Conduct superpixel segmentation on hyperspectral dataset;  3:Generate the initial solution z and calculate its objective function value F(z) using Equation (11);  4:$z^{*}\leftarrow z$;  5:**while** t<Iter **do**  6:    Perform destruction reconstruction operations in III-*B* to generate the solution z;  7:    Perform local search operations in III-*C* to generate the solution $z^{\prime }$;  8:    **if** $F\left(z^{\prime }\right)>F\left(z\right)$ **then**  9:        $z\leftarrow z^{\prime }$;10:        Update the optimal solution $z^{*}$;11:    **else**12:        Randomly generate a number *r* from 0 to 1;13:        **if** $r<e^{-\frac{\Delta F}{T}}$ **then**14:            $z\leftarrow z^{\prime }$;15:        **end if**16:    **end if**17:    T←0.99×T;18:
**end while**
19:Generate band subset *Y* according to the solution $z^{*}$;20:**return** *Y*


### 2.3. Initialization of the Solution

In SSIGA, the solution is represented as a vector $z=(z_{1},z_{2},\ldots ,z_{m},\ldots ,z_{M})$, where $z_{m}$ indicates an integer denoting the band index. For the initialization of the solution, the simplest way is random initialization, where *M* different bands are randomly selected from all the bands to form the initial solution. However, random initialization is prone to causing a heuristic algorithm to fall into local minima and can also delay its convergence. To construct a superior initial solution using the spectral information of the target HSI, we first perform clustering on all bands and then select a representative band from each cluster to form the initial solution of SSIGA.

Specifically, to effectively cluster all bands of the target HSI, commonly used clustering methods in the existing literature include subspace clustering [25], fuzzy clustering [54], density peak-based clustering [55], and deep clustering [56]. However, the number of samples contained in each of the clusters produced by these clustering methods can vary significantly. This is not conducive to constructing superior initial solutions by selecting representative bands from each cluster, and it is difficult to provide effective support for the subsequent local search strategy based on clustering results in this study. To make all clusters have a relatively balanced size, we use the *K*-means algorithm with a balanced cluster size constraint [57] to perform clustering on the target HSI. Based on the set of clusters obtained $C=\{C_{1},C_{2},\ldots ,C_{m},\ldots ,C_{M}\}$, where $C_{m}$ denotes the set of band indexes, from each group $C_{m}$ we select a band closest to the centroid of $C_{m}$ as a representative band and then use the index of the selected band as an element of the initial solution vector z. Since each of the selected bands effectively captures the overall characteristics of the corresponding cluster, the resulting initial solution ensures a certain degree of superiority.

### 2.4. Destruction and Reconstruction Operator

**Figure 2:**
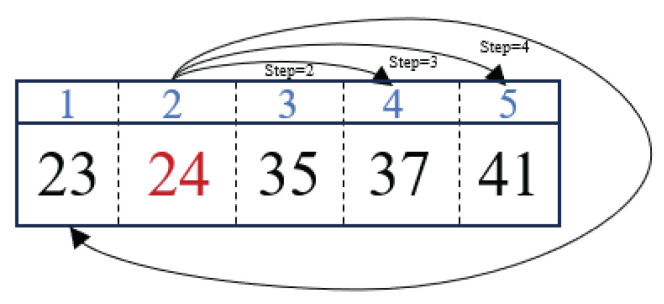
Illustration of the Levy flight strategy.

As previously mentioned, the perturbation operator in IGA is used to destroy the current solution and generate a new one. This allows IGA to expand its exploration of the solution space, thus increasing its ability to get rid of locally optimal solutions and the probability of finding a globally optimal solution. In this study, the perturbation operator of SSIGA consists of two stages: destruction and reconstruction. Let $z=(z_{1},z_{2},\ldots ,z_{m},\ldots ,z_{M})$ denote the current solution, where *M* represents the length of the solution. Consider that in the context of band selection, the sequence of selected bands does not influence the band selection outcome. Hence, during the destruction phase, two bands are randomly removed from the current solution of SSIGA. In the reconstruction stage, we select another two bands to replace the bands that are deleted in the destruction stage. Specifically, assuming that $z_{m}$ is deleted, we select a new band from the cluster where $z_{m}$ is located following the Levy flight strategy. This can be expressed as

(2) $$l^{\mathrm{new}}=l^{\mathrm{old}}+\mathrm{Levy}\left(\lambda \right),$$

where operator Levy(λ) is used to generate a step and λ denotes the stability index that can affect the value of the step [58]; $l^{old}$ indicates the serial number of the removed $z_{m}$ in the corresponding cluster, and $l^{\mathrm{new}}$ refers to the serial number of the selected band in the same cluster used to replace $z_{m}$ in the reconstruction stage. To illustrate this process more clearly, we give an example, as shown in Figure 2. In Figure 2, let $z_{m}=24$, indicating the removed band, while $l^{\mathrm{old}}=2$. To select a new band, we execute the Levy flight strategy according to Equation (2) in the cluster that band 24 belongs to (i.e., cluster $C_{1}$). In Equation (2), the operator Levy(λ) generates a random step, which represents the position of the target band after moving from the current band according to the size of the step. For example, in Figure 2, if the random steps are 2, 3, and 4, respectively, the corresponding value of $l^{\mathrm{new}}$ equals 4, 5, and 1, respectively. It should be noted that if the value of $l^{\mathrm{new}}$ exceeds the number of bands in the current cluster, it is wrapped around to fall within the valid range using the modulo operation. Moreover, Figure 2 also illustrates that when $l^{\mathrm{new}}$ equals 2, 3, and 4, respectively, we choose bands 37, 41, and 23, respectively, to replace $z_{m}$ in the reconstruction stage.

### 2.5. Spectral Information-Based Local Search

In heuristic optimization algorithms, a local search strategy can be adopted to efficiently locate improved solutions. In other words, the purpose of local search is to iteratively identify candidate solutions in the vicinity of the current solution to find a superior solution. To this end, we design a structured neighborhood-based local search strategy for SSIGA. This is achieved by first performing clustering on all bands and then constructing a *K*-NNG for each cluster. Specifically, consider the set of clusters $C=\{C_{1},C_{2},\ldots ,C_{m},\ldots ,C_{M}\}$ given by the *K*-means with a balanced cluster size constraint. For each cluster $C_{m}$, we construct a *K*-NNG $G_{m}=\left(V_{m},E_{m}\right)$, where $V_{m}=C_{m}$ and $E_{m}$, respectively, denote the vertex set and the edge set of $G_{m}$, according to the following procedure: for each band index $x_{i}$ in $V_{m}$, we generate a group of edges for $G_{m}$ by finding its *K* nearest bands within $V_{m}$ according to the Euclidean distances between two bands. The constructed set of *K*-NNGs can act as the structured neighborhoods used to guide the local search in SSIGA.

**Figure 3:**
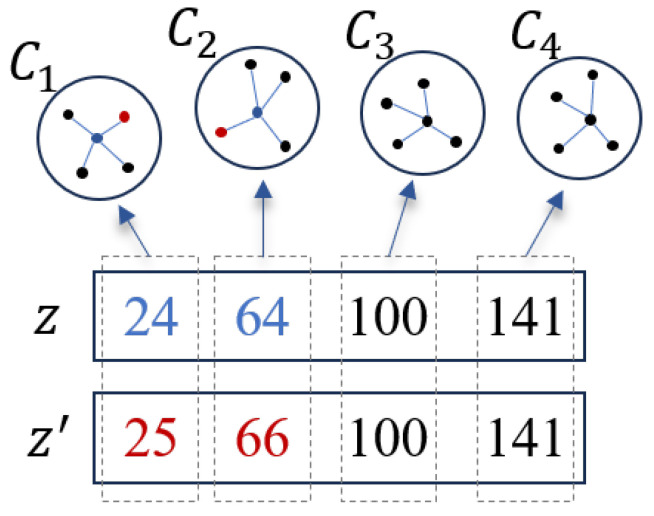
Illustration of the local search strategy.

Given the current solution $z=(z_{1},z_{2},\ldots ,z_{m},\ldots ,z_{M})$, the local search starts by randomly selecting *q* bands that will be replaced by better bands. Then, for each band $z_{m}$ of the *q* selected bands, the local search iteratively finds its adjacent bands through a predefined *K*-NNG where $z_{m}$ is located to form a new solution. To test the quality of the new solution, the objective function value corresponding to this solution is calculated according to the objective function given in Section 2.6. If the new solution is better than the current solution, the current solution is replaced with the new solution; otherwise, the current solution remains unchanged. To understand this process more intuitively, we give an example, shown in Figure 3. As shown in Figure 3, the current solution is represented as z=(24,64,100,141). According to the local search process, band 24 is first removed from the current solution; subsequently, each of its neighboring bands is selected to replace band 24 according to the *K*-NNG $G_{1}$ in which band 24 is located, and the resulting new solution is evaluated. The evaluation is based on the objective function value of the new solution. Accordingly, band 25 is identified as the best replacement band. According to this replacing strategy, band 64 is also replaced by its neighboring band 66. This process results in an updated solution $z^{\prime }=\left(25,66,100,141\right)$. It is worth noting that the proposed local search strategy has three major advantages: (1) it limits the size of the solution space to a more searchable scale; (2) it exploits the inherent spectral correlation between neighboring bands; and (3) it greatly enhances the local search capability of SSIGA, especially in terms of accelerating the convergence speed and improving the quality of the solution.

### 2.6. Objective Function Based on Spatial–Spectral Prior

Although spectral information in hyperspectral images (such as band-to-band similarity) is often used for band selection, spatial dimension features are still severely neglected. To effectively exploit the spatial information in the data, our method utilizes superpixel segmentation to capture the spatial structural information of the data. Specifically, superpixel segmentation identifies spectrally homogeneous regions to effectively capture spatial information, including texture, edges, and geometric patterns. Based on the spatial regions obtained through superpixel segmentation, we can calculate the inter-class and intra-class scattering matrices within these regions and use Fisher scores to quantitatively analyze the discrimination ability of the selected bands. Aiming to maximize discriminability and information content while minimizing redundancy of the selected band subset, we design an objective function based on the Fisher discriminant score, information entropy, and mutual information. Specifically, to measure the discriminability of the band subset selected by the current solution, we compute its Fisher discriminant score in the objective function. To this end, we first use the entropy rate superpixel (ERS) method to perform superpixel segmentation on the target HSI. Let $SP=\{sp_{1},sp_{2},\ldots ,sp_{n},\ldots ,sp_{N}\}$ represent the obtained set of superpixels, where $sp_{n}=\left\{x_{1}^{n},x_{2}^{n},\ldots ,x_{u_{n}}^{n}\right\}$ and $x_{i}^{n}$ represents the *i*-th pixel within the *n*-th superpixel. The Fisher discriminant score for the band subset selected by the current solution is expressed as

(3) $$S_{b}=\sum_{i=1}^{N}u_{i}\left(w_{i}-w\right){\left(w_{i}-w\right)}^{T}$$

(4) $$S_{w}=\sum_{i=1}^{N}\sum_{j=1}^{u_{i}}\left(x_{j}^{i}-w_{i}\right){\left(x_{j}^{i}-w_{i}\right)}^{T}$$

where $u_{i}$ indicates the total number of pixels within the *i*-th superpixel; $S_{b}$ denotes the between-class scatter matrix, measuring the dispersion of data points across different classes, and $S_{w}$ represents the within-class scatter matrix, quantifying the dispersion of data points within the same class; and *w* represents the mean vector computed across all superpixels within the target HSI, whereas $w_{i}$ signifies the mean vector specific to the *i*-th superpixel. In the context of classification tasks, a large $S_{b}$ signifies effective separation between classes, whereas a small $S_{w}$ implies tight clustering within classes. Thus, based on these metrics, the final discriminability criterion for the selected band subset is defined as

(5) $$J=\frac{\mathrm{trace}(S_{b})}{\mathrm{trace}(S_{w})}$$

where trace(·) denotes the trace of the matrix. Note that the discriminability criterion *J* ensures that the selected band subset has high between-class scatter and low within-class scatter, effectively evaluating the discriminability of the candidate band subset.

In addition, we quantify the information content of the band subset selected by the current solution through information entropy, and we assess redundancy within this subset by utilizing mutual information. In other words, information entropy, which measures the average level of uncertainty or randomness in the outcomes of a random variable, is utilized here to quantify the amount of information in all bands within the selected subset. Moreover, mutual information, which quantifies the amount of information that one random variable contains about another, is used to assess redundancy and dependencies between bands within the selected subset. Specifically, the information entropy H(x) for band *x* is calculated by [59]

(6) $$H\left(x\right)=-\sum_{\alpha \in \Phi_{x}}p\left(\alpha \right)\log p\left(\alpha \right)$$

where α denotes the gray value of band *x* and $\Phi_{x}$ represents the set of gray levels for band *x*; p(α) denotes the probability distribution of gray value α in band *x*.

Given two bands $x_{1}$ and $x_{2}$, mutual information $MI(x_{1};x_{2})$ measures the shared information between them and is calculated by [60]

(7) $$MI\left(x_{1};x_{2}\right)=H\left(x_{1}\right)+H\left(x_{2}\right)-H\left(x_{1},x_{2}\right)$$

with

(8) $$H\left(x_{1},x_{2}\right)=-\sum_{\alpha \in \Phi_{x_{1}}}\sum_{\beta \in \Phi_{x_{2}}}p\left(\alpha ,\beta \right)\log p\left(\alpha ,\beta \right)$$

where $H(x_{1},x_{2})$ denotes the joint entropy of bands $x_{1}$ and $x_{2}$.

It should be noted that according to Equations (6) and (7), a high value of information entropy means that the uncertainty of the random variable is stronger, which also means that the amount of information contained in all bands within the selected band subset is higher. In addition, a lower mutual information value signifies a weaker dependency between the variables, signifying a reduced redundancy in the selected band subset for hyperspectral classification. Therefore, maximizing the information entropy of the selected band subset while minimizing its mutual information ensures that all bands in the selected band subset have a higher level of information content and a low level of redundancy. To this end, we calculate the information entropy and mutual information of the selected band subset, respectively, by

(9) $$H^{*}=\frac{1}{M}\sum_{i=1}^{M}H\left(x_{i}\right)$$

and

(10) $$MI^{*}=\frac{2}{M^{2}-M}\sum_{1\leq i<j\leq M}MI\left(x_{i};x_{j}\right).$$

Based on Equations (5)–(10), the final objective function of SSIGA is formulated as follows:

(11) $$F=\theta \cdot J+\frac{H^{*}}{MI^{*}+\epsilon },$$

where θ denotes the weight parameter, which is empirically set to 0.002 before training and held fixed throughout optimization, and ϵ is a numerical stability term. It is worth pointing out that by maximizing the objective function in Equation (11), SSIGA intends to identify a subset of bands with high discriminability, a higher level of information content, and low redundancy.

## 3. Experiments and Discussion

In this section, we perform experimental validation of the proposed method. Specifically, we first introduce the datasets used in the experiments, including the Indian Pines, Botswana, and Kennedy Space Center (KSC) datasets. Next, we describe the experimental setup, including the parameter settings of the algorithm and the classifiers used. Subsequently, we present existing state-of-the-art methods adopted for comparative evaluation. Finally, we carry out experiments to verify the effectiveness of our method, followed by a comprehensive analysis of the experimental results.

### 3.1. Datasets

In this section, three publicly available datasets, namely Indian, Botswana, and KSC, are used to test the performance of our method (available at https://www.ehu.eus/ccwintco/index.php/Hyperspectral_Remote_Sensing_Scenes, accessed on 1 September 2025).

#### 3.1.1. Indian Pines

The Indian Pines dataset was collected by an Airborne Visible/Infrared Imaging Spectrometer (AVIRIS) sensor over the Indian Pines region in Indiana, USA. The original image contains 224 spectral channels. Each band of this image, consisting of 145 × 145 pixels, exhibits a spatial resolution of 20 m per pixel, and the wavelengths of all bands range from 400 to 2500 nm.

**Figure 4:**
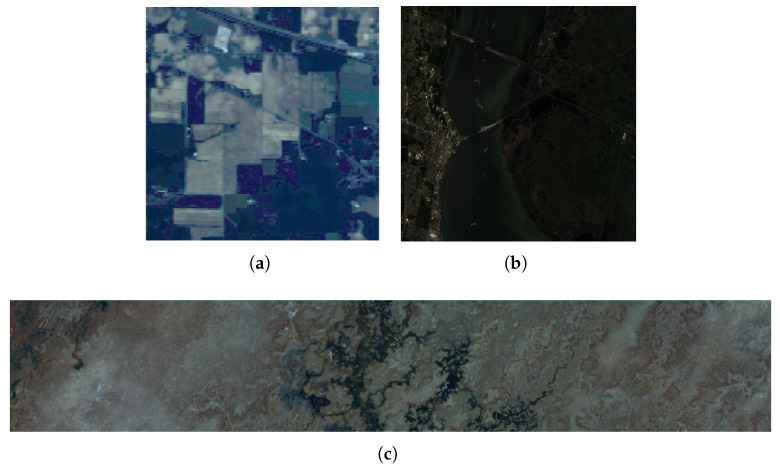
False-color composite images. (**a**) Indian Pines dataset. (**b**) KSC dataset. (**c**) Botswana dataset.

Before experiments, rigorous preprocessing steps, including the removal of noisy and water absorption bands, reduced the number of spectral channels from 224 to 200 for subsequent analysis. The available ground truth data were comprehensively classified into 16 different land cover classes. The false-color composite image of this dataset is shown in Figure 4a.

#### 3.1.2. Kennedy Space Center

The Kennedy Space Center (KSC) dataset was captured by NASA in 1996 at the Kennedy Space Center, Florida, USA, using AVIRIS. This dataset comprises 13 land cover classes and originally included 224 spectral bands. Following the removal of water absorption bands, the number of bands used in subsequent experiments was reduced to 176. This dataset spans a wavelength range from 400 nm to 2500 nm, with each pixel representing a spatial resolution of 18 m. The false-color image of this dataset is shown in Figure 4b.

#### 3.1.3. Botswana

The Botswana dataset was acquired in 2001 over the Okavango Delta region in Botswana by NASA’s EO-1 satellite, using its hyperion imaging spectrometer. This dataset includes 14 land cover classes and 242 spectral bands collectively covering a wavelength range of 400 to 2500 nm. Each band of this image contains 1476 × 256 pixels. After removing noisy and water absorption bands, 145 bands were used in our experiments. The false-color image of this dataset is shown in Figure 4c.

### 3.2. Experiment Setup

To classify the HSI and assess the quality of the selected bands, we employed two classifiers: Support Vector Machine (SVM) and Random Forest (RF). Note that the supervised classifiers (SVM and RF) are only used for quantitative evaluation of the selected band subset. The SSIGA algorithm performs band selection in a completely unsupervised manner, with no access to class labels during selection. For both classifiers, we applied consistent parameter settings across different datasets. Specifically, the Random Forest (RF) classifier was configured with 20 trees, a commonly used setting for balancing performance and computational efficiency. For Support Vector Machine (SVM), the Radial Basis Function (RBF) kernel was employed, with the penalty parameter *C* set to 5000 and the kernel parameter γ set to 0.5. Since both classifiers operate under supervised learning, we randomly selected 10% of each dataset for training [33,61]; Table 1, Table 2 and Table 3 present the number of training and test samples per class for the Indian Pines, Botswana, and KSC datasets. We evaluated the classification performance using overall accuracy (OA), average overall accuracy (AOA) [25,38,61], and the kappa coefficient (Kappa), which are widely adopted metrics in hyperspectral image classification. To evaluate the performance of our method, we compare it with several representative unsupervised band selection methods, including ASPS [25], DSC [54], E-FDPC [28], FNGBS [61], HLFC [62], MBBS-VC [41] and SNEA [33]. For fair comparison, all baseline methods utilize the hyperparameter configurations specified in their original works, while hyperparameters for our method are listed in Table 4. In addition, we also provide experimental results obtained by classifying HSIs using Support Vector Machine (SVM) and Random Forest (RF) classifiers across all original spectral bands (referred to as Baseline-SVM and Baseline-RF, respectively) for performance comparison. The following is a brief introduction to all comparison methods.

#### 3.2.1. ASPS

As a ranking-based BS method, ASPS achieves band selection by integrating subspace partitioning and band sorting techniques. Initially, the HSI cube is partitioned into multiple subcubes, with the objective of maximizing the ratio of inter-class to intra-class distances within each subcube. Subsequently, within each subcube, the band exhibiting the lowest noise level is selected as a representative band.

#### 3.2.2. DSC

This method belongs to the category of clustering-based BS methods. It leverages deep subspace clustering by seamlessly integrating subspace clustering techniques into a convolutional autoencoder framework. This integration enhances clustering performance and enables efficient end-to-end training.

#### 3.2.3. E-FDPC

This is a ranking-based cluster-driven hybrid band selection method. It calculates the score for each band through a weighted combination of two metrics: the normalized local density and a metric reflecting the compactness within clusters. These metrics are obtained using the density peak-based clustering algorithm.

#### 3.2.4. FNGBS

As a hybrid method integrating clustering and ranking principles, FNGBS begins by dividing the HSI data cube into several spectral groups and then selects spectral bands based on local density and information entropy criteria.

#### 3.2.5. HLFC

This is a clustering-based band selection method that first divides the target HSI into several regions using superpixel segmentation. After constructing a similarity graph for each superpixel to learn low-dimensional hidden features, all latent features are fused to form a unified feature representation. Finally, *K*-means is used to generate multiple clusters, from which the representative spectral band with the highest information entropy is selected.

#### 3.2.6. MBBS-VC

This is a searching-based BS method that models the BS problem as a multi-task optimization problem. It employs clustering that allows for clusters of different sizes and adopts a multi-task multi-swarm bee colony strategy, aiming to identify multiple optimal band subsets.

#### 3.2.7. SNEA

This method belongs to the search-based BS methods. It defines the BS problem as a problem of maintaining spatial structure, which is achieved by designing two optimization objectives related to spatial structure. In addition, SNEA proposes a neighborhood grouping pairwise learning strategy for generating high-quality offspring. In this strategy, a neighborhood grouping operation is developed to divide the band space into multiple groups so as to efficiently initialize the population and generate pairwise offspring solutions guided by the grouping.

### 3.3. Experimental Results and Discussion

#### 3.3.1. Comparison with State-of-the-Art Methods

**Figure 5:**
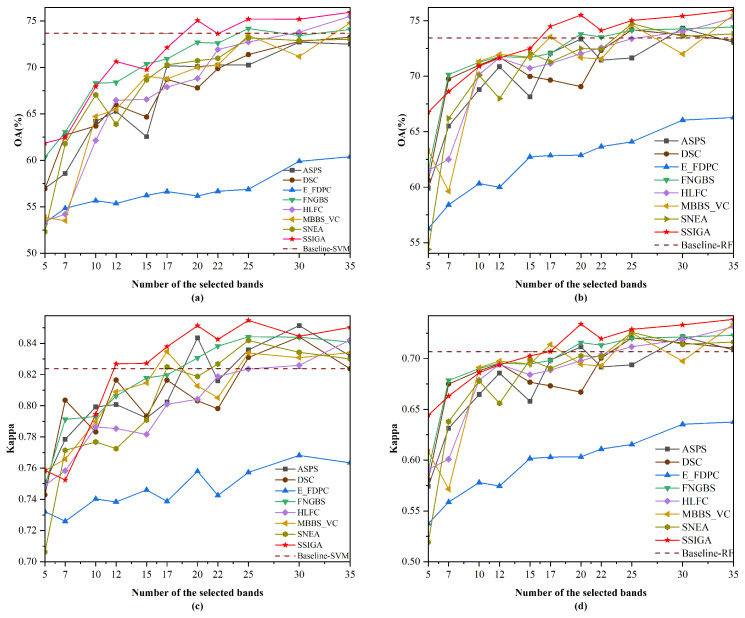
OA and Kappa versus the number of selected bands by different methods on the Indian Pines dataset. (**a**) OA obtained using the SVM classifier. (**b**) OA obtained using the RF classifier. (**c**) Kappa obtained using the SVM classifier. (**d**) Kappa obtained using the RF classifier.

**Figure 6:**
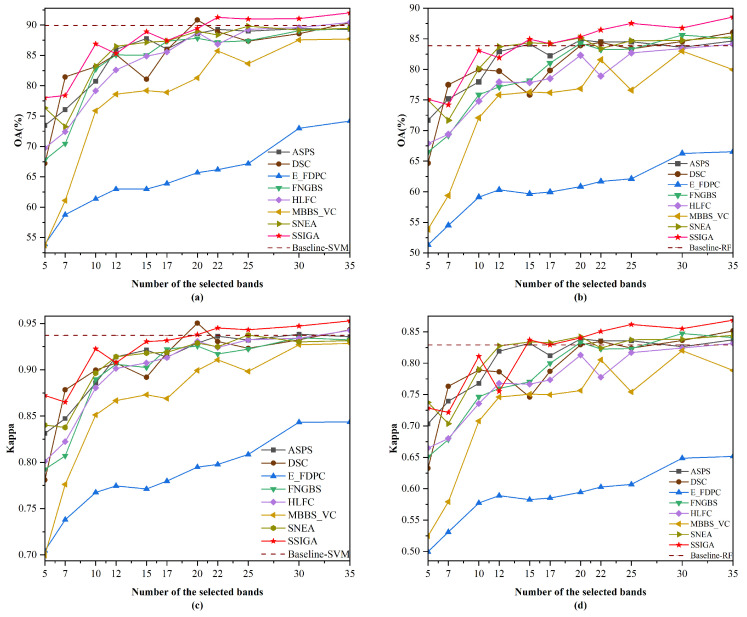
OA and Kappa versus number of selected bands by different methods on the Botswana dataset. (**a**) OA obtained using the SVM classifier. (**b**) OA obtained using the RF classifier. (**c**) Kappa obtained using the SVM classifier. (**d**) Kappa obtained using the RF classifier.

**Figure 7:**
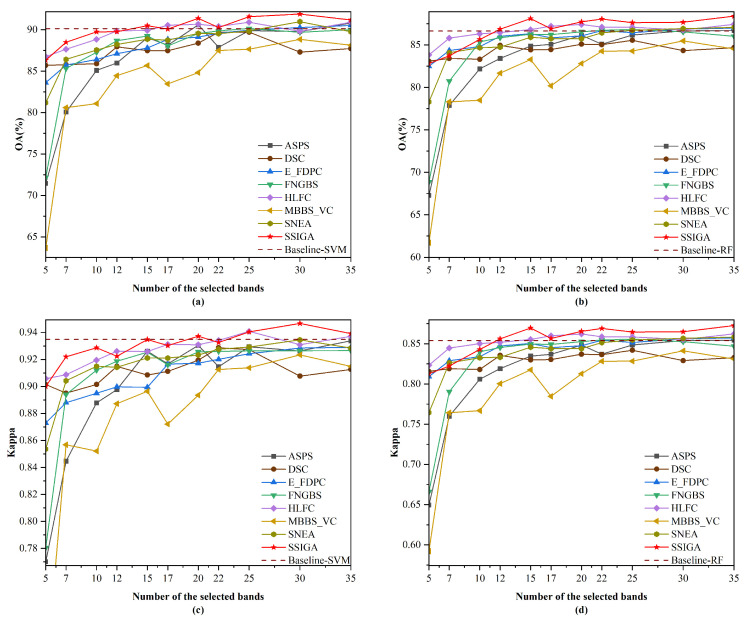
OA and Kappa versus the number of selected bands by different methods on the KSC dataset.(**a**) OA obtained using the SVM classifier. (**b**) OA obtained using the RF classifier. (**c**) Kappa obtained using the SVM classifier. (**d**) Kappa obtained using the RF classifier.

Table 5 shows the AOA and Kappa values achieved by SSIGA and seven other comparative methods using RF and SVM classifiers on Indian Pines, Botswana, and KSC datasets, where the red bold font indicates the best classification results achieved. Figure 5, Figure 6 and Figure 7 then show the OA and Kappa values obtained by all methods under different numbers of selected bands. Specifically, as shown in Table 5, the AOA values achieved by our proposed SSIGA on all three experimental datasets are higher than those achieved by the other band selection methods. In addition, SSIGA showed high consistency across all three datasets, which reflects the robustness of our method.

**Figure 8:**
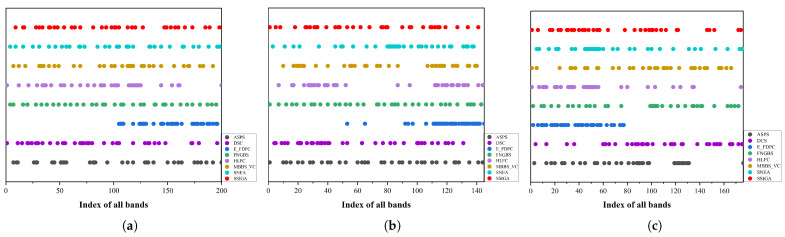
Distribution of the selected subset of bands obtained by the ASPS, DSC, E-FDPC, FNGBS, HLFC, MVPCA, MBBS-VC, and SSIGA methods. (**a**) Indian Pines. (**b**) Botswana. (**c**) KSC.

Figure 8 shows the distribution of the selected band subset by SSIGA and the other seven band selection methods on the three hyperspectral datasets. From Figure 8, it can be seen that the distribution of selected bands using different methods is relatively consistent across different datasets. The distribution of selected bands by SSIGA on Indian Pines, Botswana, and KSC datasets is more uniform in all band ranges, and the other search-based methods such as SNEA also perform well, but the distribution of selected bands for E-FDPC and HLFC is slightly concentrated. From the experimental results, it can be seen that the distribution of the selected band subsets by SSIGA on the three datasets is more uniform, indicating that the selected bands have lower redundancy and more information content.

For the Indian Pines dataset, as shown in Figure 5, SSIGA shows the best performance in most cases. Specifically, it can be seen from Figure 5a that when 5, 12, 17, 20, 22, 25, 30, and 35 bands are selected using the SVM classifier, SSIGA performs best according to the OA values. In the case that 7, 10, and 15 bands are selected, SSIGA performs slightly worse than FNGBS, but it outperforms ASPS, DSC, E-FDPC, HLFC, MBBS-VC, and SNEA. When selecting 25, 30, and 35 bands, SSIGA outperforms Baseline-SVM, further confirming that SSIGA can select a discriminative band subset. According to Figure 5b, SSIGA performs worse than DSC and FNGBS under the condition that 7 and 10 bands are used. However, when selecting 5, 15, and 35 bands with the RF classifier, SSIGA has the best OA values. In addition, SSIGA outperforms Baseline-RF at 20 to 35 bands. As can be seen from Figure 5c, at 12 to 25 and 35 bands, SSIGA gives the best Kappa values, although it shows relatively poor performance when selecting 7 bands. In addition, we can see from Figure 5d that when 5, 15, 20, and 35 bands are used, SSIGA has the best performance according to the Kappa values. Moreover, when selecting 20 to 35 bands, SSIGA outperforms the Baseline-RF method. As a whole, SSIGA achieves the best classification accuracy in most cases on the Indian Pines dataset with both classifiers.

For the Botswana dataset, as shown in Figure 6, SSIGA demonstrates the best performance in most cases. Specifically, as shown in Figure 6a, when using the SVM classifier with 5, 10, 15, 22, 25, 30, and 35 bands selected, SSIGA achieves the best performance according to the OA values. In particular, when selecting 22, 25, 30, and 35 bands, the performance of SSIGA significantly exceeds that of Baseline-SVM. In the case of selecting 7 and 20 bands, the performance of SSIGA is inferior to that of DSC and Baseline-SVM but superior to that of other methods. According to Figure 6b, when using the RF classifier, SSIGA outperformed all of the comparison methods when selecting 15, 22, 25, 30, and 35 bands. At 17 and 20 bands, SSIGA performs similarly to SNEA and outperforms other methods. It is worth noting that when selecting 15 to 35 bands, the performance of SSIGA exceeds that of the Baseline-RF method. As can be seen from Figure 6c, SSIGA obtains the best Kappa values when selecting 22 to 35 bands using the SVM classifier. At 5, 10,15, and 17 bands, the performance of SSIGA was lower than that of the Baseline-SVM method but superior to that of other methods. Moreover, from Figure 6d, it can be observed that when 22, 25, 30, and 35 bands are selected, SSIGA significantly outperforms other methods on the RF classifier according to the Kappa values. When selecting 15 and 20 bands, the performance of SSIGA is comparable to that of the SNEA and ASPS methods but superior to other methods. In general, on the Botswana dataset, SSIGA achieves the best classification accuracy in most cases with both classifiers.

For the KSC dataset, Figure 7 shows the performance of SSIGA and all methods with the SVM and RF classifiers, respectively. Specifically, we can see from Figure 7a that when selecting 15, 20, 25, 30, and 35 bands, the OA values of SSIGA are higher than those of other methods. Although SSIGA’s performance is lower than Baseline-SVM when using 7 and 10 bands, its performance exceeds that of other methods. At 22 bands, the OA value of SSIGA is comparable to that of HLFC and Baseline-SVM but higher than that of other methods. As shown in Figure 7b, when using the RF classifier to select 12, 15, 20, 22, 25, 30, and 35 bands, SSIGA demonstrates the best performance according to the OA values. At 17 bands, SSIGA performs slightly inferiorly to HLFC yet outperforms the other methods. According to Figure 7c, when 20, 30, and 35 bands are selected, SSIGA achieves the best Kappa values on the SVM classifier. When selecting 7 and 12 bands, SSIGA’s Kappa values are lower than those of Baseline-SVM but still superior to the other methods. At 15 bands, the OA value of SSIGA is comparable to that of Baseline-SVM but higher than that of other methods. When using 17 and 22 bands, the performance of SSIGA is comparable to HLFC and slightly lower than Baseline-SVM, but its performance is superior to other methods. Furthermore, as shown in Figure 7d, when selecting 12, 15, and 20 to 35 bands, SSIGA achieves the best performance on the RF classifier according to the Kappa values. At 17 bands, SSIGA performs slightly worse than HLFC but still outperforms the other methods. When 10 bands are chosen, the performance of SSIGA is inferior to that of HLFC and Baseline-RF, yet it still outperforms the other methods. Overall, on the KSC dataset, SSIGA achieves the best performance in most cases with both classifiers.

**Figure 9:**
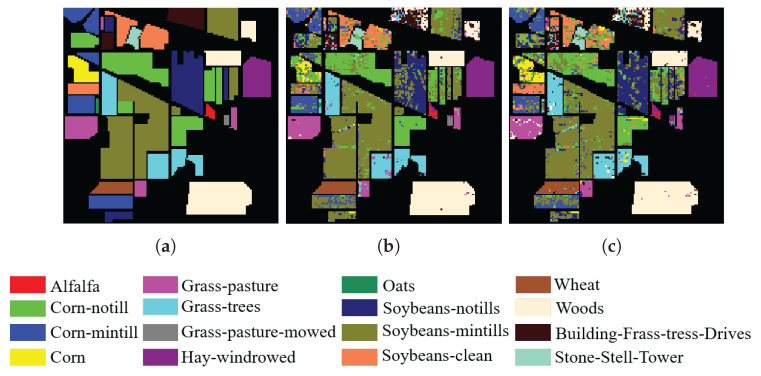
Ground truth map and classification maps of SSIGA on the Indian Pines dataset. (**a**) Ground truth. (**b**) Using SVM. (**c**) Using RF.

**Figure 10:**
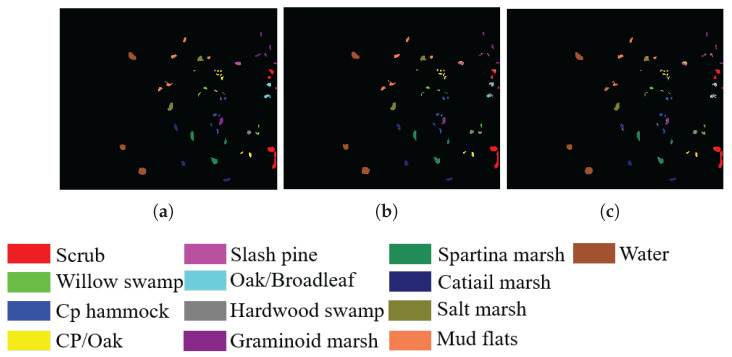
Ground truth map and classification maps of SSIGA on the KSC dataset. (**a**) Ground truth. (**b**) Using SVM. (**c**) Using RF.

**Figure 11:**
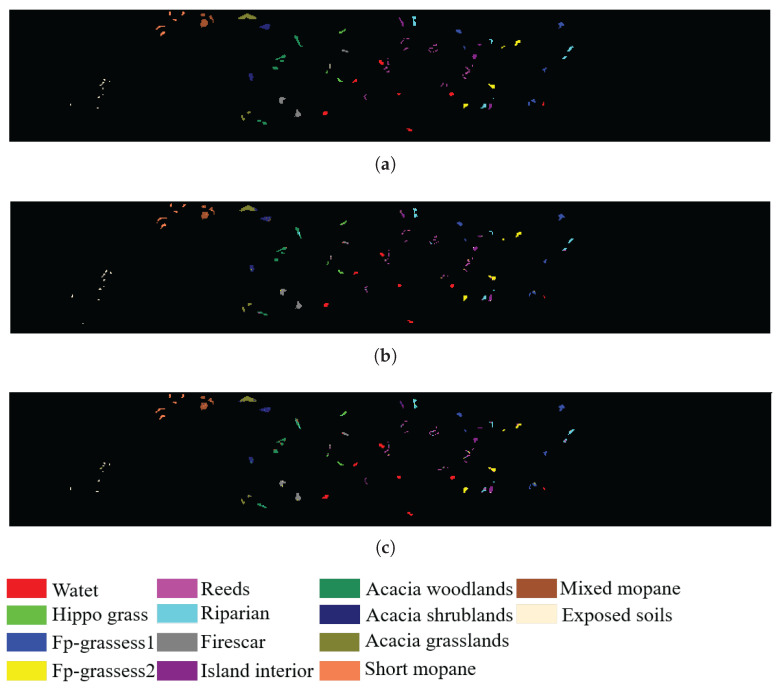
Ground truth map and classification maps of SSIGA on the Botswana dataset. (**a**) Ground truth. (**b**) Using SVM. (**c**) Using RF.

In addition, to visualize the quality of the bands selected by SSIGA, Figure 9, Figure 10 and Figure 11 give the classification maps obtained by the RF and SVM classifiers when 30 bands are selected using SSIGA on the Indian Pines, Botswana, and KSC datasets, respectively. By comparing the ground truth maps with the corresponding classification maps provided by RF and SVM classifiers, as shown in Figure 9, Figure 10 and Figure 11, we can see that SSIGA exhibits satisfactory results under the condition of removing 85%, 79%, and 83% of the bands from the Indian Pines, Botswana, and KSC datasets, respectively.

#### 3.3.2. Ablation Study

To investigate the individual impact of the local search module and the Fisher score component within the objective function, experiments with three configurations were carried out on the three datasets. Three ablation experimental settings were used in our experiment: SSIGA without local search, SSIGA without Fisher score, and complete SSIGA. Specifically, in the first setting, the local search module in SSIGA was disabled, the band neighborhood structure was removed, and only destruction and reconstruction were applied to the solution during algorithm iteration. In the second setting, the Fisher score was removed from the objective function of SSIGA, while the information entropy and mutual information components were retained. In the third setting, the complete SSIGA was used.

The detailed results are presented in Table 6, where the red bold font indicates the best classification results achieved and the symbol ✓ indicates that the corresponding module is enabled. As shown in Table 6, disabling either the local search module or the Fisher score component degrades the classification performance of SSIGA to varying degrees. Specifically, the absence of local search has a more pronounced impact, likely because the band neighborhood-based local search enhances the optimization capability of SSIGA, whereas destruction and reconstruction alone cannot explore the solution space effectively. Meanwhile, the removal of the Fisher score also led to a performance drop, demonstrating its effectiveness in capturing and utilizing spectral information, even though the remaining components (information entropy and mutual information) still provide a guiding function for the search.

#### 3.3.3. Execution Time

Table 7 reports the executing time of our proposed SSIGA method and other comparative methods, with the best results highlighted in red bold font. From Table 7, we can see that SSIGA runs longer than ASPS, DSC, E-FDPC, FNGBS, and SNEA on the Indian pine dataset but shorter than HLFC and MBBS-VC. On the Botswana and KSC datasets, SSIGA took more time than ASPS, E-FDPC, FNGBS, HLFC, and SNEA but less time than DSC and MBBS-VC. Overall, our proposed SSIGA requires considerable computation time to iteratively search for the optimal band subset. Nevertheless, it can select band subsets that yield significantly more accurate classification performance compared to other methods.

## 4. Conclusions

In this paper, we have proposed a novel unsupervised BS method, termed Spectral–Spatial Iterative Greedy Algorithm-based band selection (SSIGA). Our method introduces clustering and superpixel segmentation techniques into the iterative greedy algorithm, thereby enhancing the efficiency and effectiveness of band selection by leveraging both spectral and spatial characteristics of the data. By employing a *K*-means clustering method with balanced cluster size constraints, we have facilitated efficient local search using spectral information. The construction of *K*-nearest neighbor graphs for each cluster has further enabled the generation of high-quality neighborhood solutions. In addition, we have designed an effective objective function that evaluates the discriminability and information redundancy of the selected bands. This function leverages superpixel segmentation to calculate the Fisher score for each band, as well as the average information entropy and mutual information among the bands, thus providing a comprehensive assessment of the solution quality. Experimental results on three publicly available real HSI datasets have demonstrated the superiority of the SSIG method compared to several state-of-the-art methods.

## Figures and Tables

**Table 1:** Number of training and testing samples in the Indian Pines dataset.

Class	Name	Training Samples	Testing Samples
1	Alfalfa	5	41
2	Corn-notill	143	1285
3	Corn-mintill	83	747
4	Grass-pasture	24	213
5	Corn	49	434
6	Grass-trees	73	657
7	Grass-pasture-mowed	3	25
8	Hay-windrowed	48	430
9	Oats	2	18
10	Soybeans-notills	98	874
11	Soybeans-mintills	246	2209
12	Soybeans-clean	60	533
13	Wheat	21	184
14	Woods	127	1138
15	Building-Grass-Tress-Drives	39	347
16	Stone-Steel-Tower	10	83

**Table 2:** Number of training and testing samples in the Botswana dataset.

Class	Name	Training Samples	Testing Samples
1	Water	27	243
2	Hippo grass	11	90
3	Fp-grassess1	26	225
4	Fp-grassess2	22	193
5	Reeds	27	242
6	Riparian	27	242
7	Firescar	26	233
8	Island interior	21	182
9	Acacia woodlands	32	282
10	Acacia shrublands	25	223
11	Acacia grasslands	31	274
12	Short mopane	19	162
13	Mixed mopane	27	241
14	Exposed soils	10	85

**Table 3:** Number of training and testing samples in the Kennedy Space Center dataset.

Class	Name	Training Samples	Testing Samples
1	Scrub	77	684
2	Willow swamp	25	218
3	CP hammock	26	230
4	CP/Oak	26	226
5	Slash pine	17	144
6	Oak/Broadleaf	23	206
7	Hardwood swamp	11	94
8	Graminoid marsh	44	387
9	Spartina marsh	52	468
10	Catiail marsh	41	363
11	Salt marsh	42	377
12	Mud flats	51	452
13	Water	93	834

**Table 4:** Values of the hyperparameters.

Parameters	Values
Simulate annealing initial temperature, *T*	1000
Maximum numuber of iterations, iter	2000
Number of superpixels, *N*	300

**Table 5:** Results obtained by the proposed SSIGA and other methods using RF and SVM classifiers on Indian Pines, Botswana, and KSC datasets (%).

Dataset	Classifier	SSIGA	ASPS [25]	DSC [54]	E-FDPC [28]	FNGBS [61]	HLFC [62]	MBBS-VC [41]	SNEA [33]
Indian Pines	AOA(RF)	**72.80**	69.91	70.47	62.13	71.68	70.44	70.61	69.91
	Kappa(RF)	**70.46**	67.63	68.19	59.59	69.45	68.18	68.36	67.64
	AOA(SVM)	**70.88**	66.69	67.07	56.54	69.85	66.68	66.82	67.70
	Kappa(SVM)	**82.19**	80.93	80.50	74.64	81.59	79.78	80.80	79.94
Botswana	AOA(RF)	**83.45**	81.45	79.97	60.21	79.02	77.98	73.78	82.02
	Kappa(RF)	**81.44**	80.43	78.91	58.81	77.93	76.83	72.57	81.02
	AOA(SVM)	**87.25**	85.05	84.53	64.55	83.57	83.57	77.57	85.42
	Kappa(SVM)	**92.32**	90.76	90.50	78.40	89.56	89.91	86.36	90.71
KSC	AOA(RF)	**86.68**	82.85	84.39	85.71	84.18	86.56	80.56	85.11
	Kappa(RF)	**85.42**	81.34	82.95	84.37	82.75	85.29	78.79	83.73
	AOA(SVM)	**90.11**	86.25	87.55	88.09	87.27	89.66	83.26	88.24
	Kappa(SVM)	**93.03**	89.79	91.16	90.81	90.70	92.64	87.33	91.56

Red bold font indicates the best results.

**Table 6:** Ablation study on the “local search” and “Fisher score” components.

Dataset	Local Search	Fisher score	AOA	Kappa
Indian Pines		✓	64.93	76.98
	✓		68.33	79.27
	✓	✓	**70.88**	**82.19**
Botswana		✓	83.06	89.97
	✓		85.41	90.15
	✓	✓	**87.25**	**92.32**
KSC		✓	86.39	90.01
	✓		89.08	92.29
	✓	✓	**90.11**	**93.03**

Red bold font indicates the best results.

**Table 7:** The executing time (in seconds) spent by SSIGA and other methods when selecting 10 bands on the Indian Pines, Botswana, and KSC datasets.

Dataset	ASPS	DSC	E-FDPC	FNGBS	HLFC	MBBS-VC	SNEA	SSIGA
Indian Pines	0.11	14.27	0.29	**0.05**	55.44	62.90	4.80	30.58
Botswana	1.22	206.07	0.58	**0.45**	41.51	1224.76	5.72	138.93
KSC	1.24	208.97	0.62	**0.47**	52.56	1257.03	5.88	155.70

Red bold font indicates the best results.

## Data Availability

Data are contained within the article.

## Abbreviations

The following abbreviations are used in this manuscript:

BS	Band Selection
HSIs	Hyperspectral remote sensing images
SSIGA	Spectral–Spatial Iterative Greedy Algorithm
K-NNGs	K-Nearest Neighbor Graphs
IGA	Iterative Greedy Algorithm
ERS	Entropy Rate Superpixel
KSC	Kennedy Space Center
ASPS	Adaptive Subspace Partition Strategy
DSC	Deep Subspace Clustering
E-FDPC	Enhanced Fast Density-Peak-based Clustering
FNGBS	Fast Neighborhood Grouping for Band Selection
HLFC	Hierarchical Latent Feature Clustering
MBBS-VC	Multi-task Bee Band Selection With Variable-Size Clustering
SNEA	Structure-Conserved Neighborhood-Grouped Evolutionary Algorithm
SVM	Support Vector Machine
RF	Random Forest
RBF	Radial Basis Function
OA	Overall Accuracy
AOA	Average Overall Accuracy
